# Phantom investigation of 3D motion-dependent volume aliasing during CT simulation for radiation therapy planning

**DOI:** 10.1186/1748-717X-2-10

**Published:** 2007-02-24

**Authors:** James A Tanyi, Martin Fuss, Vladimir Varchena, Jack L Lancaster, Bill J Salter

**Affiliations:** 1 Department of Radiation Oncology, University of Arizona Health Science Center, Tucson, AZ 85724, USA; 2 Department of Radiation Oncology and Radiation Medicine, Oregon Health and Science University, Portland, OR 97239, USA; 3 Department of Radiation Oncology, University of Texas Health Science Center at San Antonio, San Antonio, TX 78229, USA; 4 Computerized Imaging Reference Systems (CIRS), Incorporated, Norfolk, VA 23513, USA; 5 Research Imaging Center, University of Texas Health Science Center at San Antonio, San Antonio, TX 78284, USA; 6 Department of Radiation Oncology, University of Utah/Huntsman Cancer Institute, Salt Lake City, UT 84112, USA

## Abstract

**Purpose:**

To quantify volumetric and positional aliasing during non-gated fast- and slow-scan acquisition CT in the presence of 3D target motion.

**Methods:**

Single-slice fast, single-slice slow, and multi-slice fast scan helical CTs were acquired of dynamic spherical targets (1 and 3.15 cm in diameter), embedded in an anthropomorphic phantom. 3D target motions typical of clinically observed tumor motion parameters were investigated. Motion excursions included ± 5, ± 10, and ± 15 mm displacements in the S-I direction synchronized with constant displacements of ± 5 and ± 2 mm in the A-P and lateral directions, respectively. For each target, scan technique, and motion excursion, eight different initial motion-to-scan phase relationships were investigated.

**Results:**

An anticipated general trend of target volume overestimation was observed. The mean percentage overestimation of the true physical target volume typically increased with target motion amplitude and decreasing target diameter. Slow-scan percentage overestimations were larger, and better approximated the time-averaged motion envelope, as opposed to fast-scans. Motion induced centroid misrepresentation was greater in the S-I direction for fast-scan techniques, and transaxial direction for the slow-scan technique. Overestimation is fairly uniform for slice widths < 5 mm, beyond which there is gross overestimation.

**Conclusion:**

Non-gated CT imaging of targets describing clinically relevant, 3D motion results in aliased overestimation of the target volume and misrepresentation of centroid location, with little or no correlation between the physical target geometry and the CT-generated target geometry. Slow-scan techniques are a practical method for characterizing time-averaged target position. Fast-scan techniques provide a more reliable, albeit still distorted, target margin.

## Background

Tumor localization for treatment planning in radiation oncology is commonly performed using computed tomography (CT). Owing to image matrix selection, slice thickness, and window and level settings, an overestimation of a static target's physical volume may be observed due to partial volume sampling uncertainty effects [1]. Organ motion, most pronouncedly observed in the thorax and the abdomen, further challenges CT-based targeting due to the potential for insufficient temporal sampling of the moving target. Clinically, these uncertainties can result in errors in representation of true tumor location, extent, and associated motion envelope (the three-dimensional-space that is occupied by a target volume due to respiration and other motion inducing positional variations). Thus, it is critical to understand the potential problems and limitations with CT simulation image acquisition as they correlate directly with the capability to accurately deliver a radiation oncology treatment at anatomical sites that are subject to organ motion. It should be noted that recently, so-called 4D imaging techniques have become available in radiation oncology, wherein CT scanners capable of multislice acquisition are utilized in cine-mode to acquire time-stamped projections which allow for a binned reconstruction of CT motion data. While the advent of this exciting new imaging modality holds much promise, the vast majority of CT simulation studies currently conducted in radiation oncology are still performed via non-4D, helical scanning techniques. This can be attributed both to the very recent emergence of the 4D scanning technique, along with the inherent requirement that to perform such 4D scans expensive and specialized equipment must be acquired, not the least of which would include a multi-slice-capable CT scanner. For this reason we restrict the scope of the current study to the currently, more commonly employed helical scan technique.

Volume aliasing, understood as a CT misrepresentation of the true spatial and geometric parameters of well-defined volumes, has been investigated experimentally and/or analytically for targets moving freely in a single dimension (longitudinally or transversally) [2,3]. Pertinent motion/imaging parameters that have been considered include initial motion phase, motion amplitude, and scan speed. To supplement current understanding of volume aliasing, the present study investigates the impact of clinically relevant, three-dimensional (3D) target motion of well-defined geometric targets using a prototype motion phantom (now commercially available from CIRS, Computerized Imaging Reference Systems Inc., Norfolk, VA, USA). The specific aims of this study were to (1) experimentally quantify volume aliasing for known, clinically relevant, 3D tumor motion amplitudes as a function of CT image acquisition mode (helical), and CT rotation time, and (2) to provide a qualitative understanding of 3D tumor motion effects on the accuracy of tumor localization. The data collected should provide a valuable context for the evaluation of the potential value of recently emerging 4D scanning techniques.

## Materials and methods

### Phantom description

**Figure 1:**
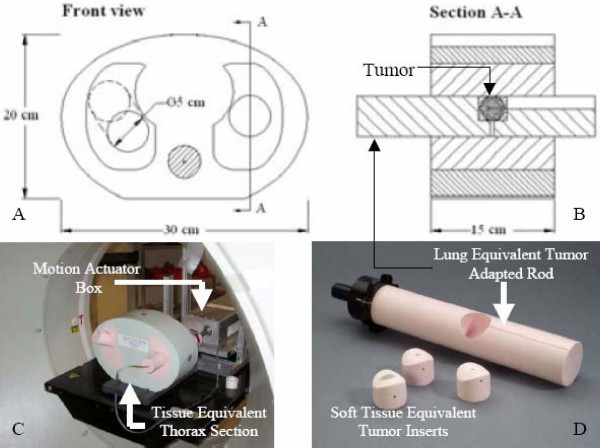
Dynamic thorax phantom designed for studies of the effect of motion on localization and characterization of moving targets during pretreatment CT. Images A and B are axial and sagittal drawings of the tissue equivalent thorax section depicted in C. Image B is a cut through the lung equivalent target adapted rod. A computer-controlled actuator applies complex three-dimensional motions to the target within the phantom body through the lung equivalent target adapted rod. S-I motion can be isolated from, or synchronized with, R-L and A-P motion in both frequency and amplitude, enabling sinusoidal and/or other complex motions to be achieved with sub-millimeter accuracy and reproducibility.

A prototype dynamic anthropomorphic thorax phantom (commercially available from CIRS Inc., Norfolk, VA, USA) was used in this study. Modifications, relevant to the conduct of the present study, regarding the original phantom specifications were designed by the investigators and implemented by the phantom vendor. The phantom (figure 1) is a 15 cm thick tissue equivalent thorax section that represents an average human thorax anatomy in shape, proportion and composition. The phantom is manufactured from lung, bone, and soft tissue equivalent materials to simulate the heterogeneous environment of the human thorax. Table 1 is a summary of the physical properties of the equivalent tissue materials constituent of the phantom. Lung equivalent rod subsections, 40 and 70 mm in diameter, embedded in the lung-equivalent section of the phantom, are used to house spherical, soft tissue equivalent, tumor-simulating targets of various sizes. The phantom sits on an alignment base plate that is connected to a motion actuator box. A motion actuator is used to induce target motion through the translation and rotation of the lung equivalent rod. A computer programmed motion control unit and cable assembly is used to drive the motion actuator. The center of mass, or centroid, of the available targets is positioned at an off central-axis location in the lung equivalent rod, thus facilitating three dimensional (3D) motion of the target through simultaneous rotation and translation of the lung equivalent rod. The target can describe linear motion in the longitudinal, or superior-inferior (S-I), direction of up to ± 20 mm, with an accuracy of 0.05 mm about its reference position. Rotational motion about the central axis of the tumor-adapted rod allows the centroid of the target to describe an arc ranging from 0° to 180° axially with an accuracy of 0.2°. The range of motion of the target centroid in the anterior-posterior (A-P) and the right-left (R-L) directions can be computed knowing the distance of the target centroid from the central axis of the tumor-adapted rod and the ± angle of rotation of the tumor-housing lung equivalent rod. Linear motion in the S-I direction can be isolated from rotational motion in the axial direction in both frequency and amplitude. Linear and rotational motions can be synchronized to one another with accuracy better than 20 msec, thus enabling simple sinusoidal tumor motion in 3D space. Finally, motion cycles ranging from 4 – 7 seconds, with accuracy better than 5 msec, can be programmed.

**Table 1:** Physical quantities pertaining to phantom composition.

Phantom Material	Density (g/cm^3^)	Electron Density (× 10^23 ^cm^-3^)	Relative Electron Density ρ_*e*_
Lung	0.21	0.69	0.207
Bone	1.60	5.03	1.506
Plastic Water^®^-Diagnostic/Therapy Range	1.04	3.35	1.003
Soft Tissue Target	1.06	3.43	1.028

The last column is a comparison of the relative electron densities of the various tissue equivalent materials.

### Target and motion parameters

Two spherical targets; 10 and 31.5 mm in diameter, were used in this investigation. The 10 mm (or small) target was embedded in the 40 mm diameter lung equivalent rod and the 31.5 mm (or large) target in the 70 mm rod. Clinically realistic patient breathing cycles, which may have complex patterns and non-constant amplitude and periodicity [4], were approximated by the 3D sinusoidal model described above. Both targets were programmed to execute ± 5 mm, ± 10 mm, and ± 15 mm excursions in the S-I direction about their corresponding reference positions. In addition to programmed longitudinal motion, by choosing appropriate simultaneous rotation about the longitudinal axis (S-I), clinically realistic tumor motions in both the A-P and L-R directions were also programmed (± 5 mm and ± 2, respectively, for each of the above S-I motion amplitudes). The 3D motion amplitudes programmed were selected to reflect clinically relevant tumor motions commonly observed for pulmonary lesions. Motion cycle period was set at 4 seconds, consistent with typical human breathing cycles and previously used values [5]. Data was collected for a target in a static mode (target stationary) and dynamic mode (target undergoing three-dimensional motion involving simultaneous S-I, A-P, and L-R displacements).

**Figure 2:**
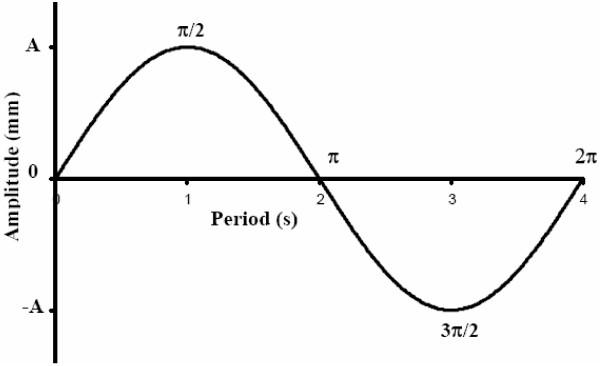
2D representation of motion of target centroid as a function of time. Motion is sinusoidal with period of 4 sec. Motion amplitude (A) represents the maximum excursion of the target centroid in the S-I direction about a reference position (0), and takes values ± 5 mm, ± 10 mm, or ± 15 mm. Motion phases π/2 and 3π/2 respectively coincide with the superior- and inferior-most excursions of the target centroid about the reference (0) position.

Because CT imaging of dynamic targets is highly motion phase dependent [2], consistent image-acquisition-to-motion-phase synchronization schemes were used in this study on all scans involving target motion. Phase was defined as the angle in sinusoidal motion at which the CT scanner beam was enabled. Phase synchronization was achieved by initiating beam-on at the same initial scan plane and identical motion phase of the target on all studies. Figure 2 is a 2D representation of the target centroid motion as a function of cycle period. Motion phase π/2 and 3π/2 respectively coincide with the superior- and inferior-most excursions of the target centroid about the reference (0) position.

### Imaging modality

A single-slice helical CT scanner (PQ 5000, Philips Medical Systems, Bothell, WA, USA) and a 4-slice multi-slice helical CT scanner (LightSpeed™ RT, GE Medical Systems, Milwaukee, WI, USA) were used for image acquisition. Axial CT imaging is beyond the scope of this work and was not investigated. All CT scans were acquired along the couch axis in the superior to inferior direction. Display field of view was set at 450 mm and a reconstruction matrix of 512 × 512 was used. Scan parameters used were typical of thoracic simulation at the Cancer Therapy and Research Center, San Antonio, TX. These include 1.5 pitch, 120 kVp, 300 mA, and 3 mm slice thickness for the single-slice technique, and 0.75:1 pitch, 140 kVp, 205 mA and 2.5 mm slice thickness for the multi-slice technique. Fast (1 second/rotation) scan speed and a slow (4 second/rotation) speed scan techniques were used to assess the effect(s) of imaging speed and motion amplitude on volume aliasing. For each scan speed and target size, the motion amplitudes specified in Section 2.2 were systematically examined for 8 different initial target motion phases, each separated by π/4.

### Data analysis: target segmentation and aliased data generation

All studies were transferred electronically to a radiation treatment planning station (CORVUS version 5.0, North American Scientific/NOMOS, Cranberry Township, PA) where treatment planning software inherent tools were used for target volume delineation and analysis.

Target segmentation was performed on a default window/level (W = 400 HU and L = -700 HU) in the treatment planning system, as applicable to thoracic/lung structures. To eliminate user bias in delineating the target volume, a software-inherent, semi auto-segmentation technique was utilized to systematically define the outer boundary of the target as the most peripheral density voxels which were readily distinguishable from background. The delineation process was confirmed to be consistent and reproducible. The contoured volume for each study involving a moving target was termed the *dynamic gross target volume *(dGTV) to distinguish it from a corresponding *static gross target volume *(sGTV) generated from a stationary target.

By summing all voxels enclosed within a segmented volume, the volumes of the dGTV and sGTV were computed. Subsequently, the stereotactic coordinates of the centroid of both the sGTV and dGTV were automatically computed by the treatment planning software.

### Benchmark volumetric information for aliasing quantification

#### 1. Volumetric misrepresentation

True physical volumes, or tTVs, of the 10 and 31.5 mm diameter targets were measured and computed (formula; see Appendix) and then compared with manufacturer reported values, with good agreement. These values were subsequently used to quantify target volume mis-estimation (over/under estimation) in the presence of motion. The mis-estimation factor was computed as a ratio of the dGTV to its corresponding known volume (tTV). Mis-estimation factors were not computed for sGTVs (i.e. due to partial volume effects) as this has been extensively investigated by Winer-Muram and colleagues [1].

Time-averaged motion envelopes were mathematically computed (formula; see Appendix) for each target for three known motion amplitudes. The quantitative values of the motion envelopes (here referred to as tGTV) were used to analyze the degree to which each dGTV approximated its corresponding motion envelope, reported as the ratio of a dGTV over its corresponding (true) motion envelope.

#### 2. Reference centroid misplacement

The location of each delineated structure (sGTV or dGTV) was defined by its geometric center, or centroid. The reference centroid position was defined using scan parameters in Section 2.3, with each target stationary at its reference position. To quantify the degree of misinterpretation of the target location as a result of target motion, the 3D displacement vector of the various dGTV centroids were computed.

## Results

Table 2 summarizes two important parameters for the small and large targets: 1) true physical volumes, or tTVs and 2) time-averaged (true) motion envelopes for three known motion amplitudes, or tGTVs.

**Table 2:** Summary of mathematically computed benchmark quantities.

Target Diameter (mm)	True Physical Volume (tTV) (cm^3^)	Motion envelope (tGTV) (cm^3^) for known motion Amplitude (mm)		
		
		± 5 mm	± 10 mm	± 15 mm
10	0.52	2.45	2.88	3.46
31.5	16.37	34.96	39.34	45.20

### True target volume mis-estimation

**Figure 3:**
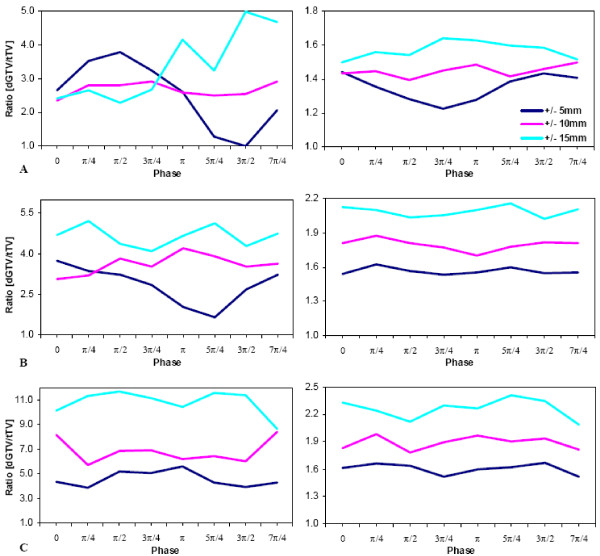
Magnitude of mis-estimation of the true physical target volumes (tTV) of the 10- and 31.5-mm diameter targets as a function of eight (8) initial motion phases for three (3) motion amplitudes. The mis-estimation magnitude is computed as a ratio of each CT reconstructed dGTV and its corresponding tTV. Plots A, B, and C correspond to single-slice fast (or 1-sec), multi-slice fast (or 1-sec), and single-slice slow (or 4-sec) scan imaging techniques, respectively. Each colored line represents a specific motion amplitude in the S-I direction, synchronized with constant amplitudes of ± 2 and ± 5 mm in the R-L and A-P, respectively.

Figure 3 is a graphical representation of the variation of the target volume mis-estimation (dGTV/corresponding tTV ratio) as a function of phase and motion amplitude during single-slice fast scan-, multi-slice fast scan-, and single-slice slow scan-CT techniques. The plots depict a general trend of target volume overestimation in the presence of target motion during CT imaging. Overall, the smaller target showed a greater percentage overestimation than the larger one.

Table 3 is a quantitative summary of Fig 3. The key findings were as follows: 1) the mean percentage overestimation of the tTV increased with target motion amplitude and decreased with increasing target diameter; 2) though slow scan techniques resulted in greater volume overestimation, slow-scan generated volumes, like fast scan generated ones, were seen to be motion-phase dependent; and 3) the small-target percentage overestimation was more susceptible to initial motion phase changes than the larger target. The mean overestimation for single-slice fast scan CT technique was as much as 3.38 times (or a 238% increase) for the small (10 mm diameter) tTV and 1.57 times (or a 57% increase) for the large (31.5 mm diameter) tTV. The mean overestimation for multi-slice fast scan CT technique was as much as 4.65 times (or a 365% increase) for the small tTV and 2.08 times (or a 108% increase) for the large tTV. Finally, the mean overestimation for single-slice slow scan CT technique was as much as 11.1 times (or a ~1000% increase) for the small tTV and 2.26 times (or a 126% increase) for the large tTV.

**Table 3:** Range of volume over/under-estimation as a function of motion amplitude for the three scan modes.

Target Diameter (mm)	S-I Motion Amplitude ± (mm)	Single-slice Fast				Multi-slice Fast				Single-slice Slow			
		
		Min	**Mean**	**1σ**	Max	Min	**Mean**	**1σ**	Max	Min	**Mean**	**1σ**	Max
	5	0.99	**2.52**	**1.02**	3.78	1.66	**2.85**	**0.70**	3.74	3.88	**4.57**	**0.62**	5.60
	10	2.35	**2.68**	**0.21**	2.92	3.07	**3.61**	**0.37**	4.2	5.71	**6.84**	**0.99**	8.42
10	15	2.27	**3.38**	**1.07**	4.98	4.11	**4.65**	**0.39**	5.21	10.1	**11.1**	**0.60**	11.7

	5	1.22	**1.35**	**0.08**	1.44	1.54	**1.56**	**0.03**	1.62	1.52	**1.60**	**0.06**	1.67
	10	1.39	**1.45**	**0.03**	1.50	1.7	**1.79**	**0.05**	1.87	1.78	**1.89**	**0.07**	1.97
31.5	15	1.50	**1.57**	**0.05**	1.64	2.02	**2.08**	**0.05**	2.15	2.09	**2.26**	**0.11**	2.41

Each target motion in the S-I direction was synchronized with a fixed rotational motion to initiate an R-L and an A-P displacement of ± 2 and ± 5 mm, respectively.

**Figure 4:**
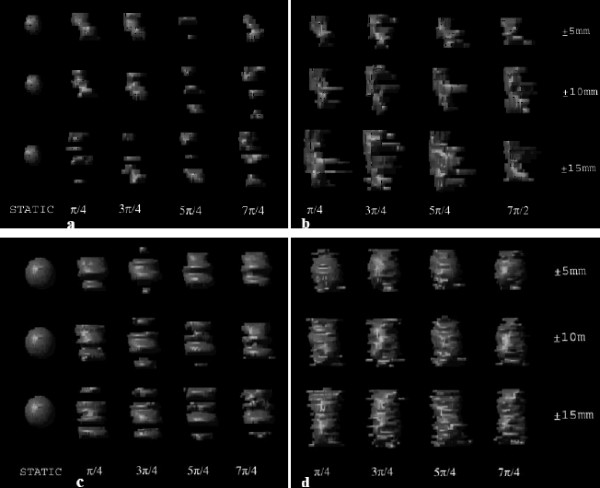
Fast- and slow-scan distortion of the 10- and 31.5-mm diameter targets as a function of four (4) initial motion phases and three (3) motion amplitudes. The top row of images ("a" and "b") is associated with the 10 mm target, while the bottom row ("c" and "d") with the 31.5 mm target. The columns of structures labeled "STATIC" are surrogate representations of the respective 10- and 31.5-mm diameter targets. Image sets "a" and "c" are reconstructions from single-slice fast techniques, while "b" and "d" are from single-slice slow scan techniques. The motion amplitudes presented on the figures are for the S-I direction and are synchronized with constant ± 2 and ± 5 mm displacements in the R-L and A-P directions, respectively.

For qualitative appreciation of motion-induced volumetric distortion during CT imaging, frontal views of the dGTVs for both the small and large targets are presented in Fig 4. It is apparent that there is little similarity between the dGTVs and the sGTV (the sGTV being a proxy representation of the true geometry of each corresponding target).

### Reproducibility of time-averaged motion envelope

Table 4 summarizes quantitatively the degree to which each dGTV approximates its corresponding motion envelope. The key results were as follows: 1) fast scan dGTVs are generally smaller in magnitude that their corresponding tGTVs, and changing target diameter from 10 mm to 31.5 mm does not result in a significant change in the dGTV/tGTV ratio. 2) Slow-scan dGTVs may either be smaller or larger than their corresponding tGTVs, depending on motion amplitude and phase. 3) Changing target diameter from 10 mm to 31.5 mm did decrease the dGTV/tGTV ratio, bringing it closer to 1.0.

**Table 4:** Ratio of dGTVs and corresponding tGTVs.

Target Diameter (mm)	S-I Motion Amplitude ± (mm)	Single-slice Fast			Multi-slice Fast			Single-slice Slow		
		
		Min	**Mean**	Max	Min	**Mean**	Max	Min	**Mean**	Max
10	5	0.21	**0.54**	0.81	0.36	**0.61**	0.80	0.83	**0.98**	1.20
	10	0.50	**0.57**	0.62	0.66	**0.77**	0.90	1.22	**1.46**	1.80
	15	0.51	**0.76**	1.07	0.88	**0.99**	1.11	2.17	**2.38**	2.50

31.5	5	0.57	**0.63**	0.67	0.72	**0.73**	0.76	0.71	**0.75**	0.78
	10	0.65	**0.68**	0.70	0.80	**0.84**	0.88	0.83	**0.88**	0.93
	15	0.70	**0.73**	0.77	0.94	**0.98**	1.01	0.98	**1.06**	1.13

Ratio gauges the proximity of the magnitude of a reconstructed dGTV with that of its corresponding time-averaged motion profile (tGTV). Each target motion in the S-I direction was synchronized with a fixed rotational motion resulting in an R-L and an A-P displacement of ± 2 and ± 5 mm, respectively.

### Phase-synchronization-related centroid misplacement

**Figure 5:**
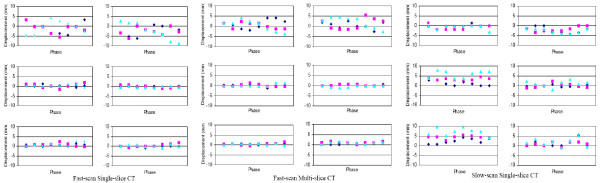
Reference centroid misplacement as a function of three (3) motion amplitudes and eight (8) initial motion phases for the 10- and 31.5-mm diameter targets. Plots were generated by reconstructing scans from single-slice fast (1-sec), multi-slice fast (1-sec), and single-slice slow (4-sec) scan techniques, respectively. The plots in the first, second and third rows represent misplacement of the reference centroid location (0) in the S-I, A-P, and L-R directions, respectively. The blue, pink and teal colored lines represent motion amplitudes in the S-I direction of ± 5, ± 10, and ± 15 mm, respectively. Each S-I motion is synchronized with an A-P and an L-R motion of ± 2 and ± 5 mm, respectively.

Figure 5 is an illustration of how much the reference centroid of stationary target (once again, a proxy representation of the true centroid of each corresponding target) is displaced if imaged while in motion. Table 5 is a quantitative summary of Fig 5. No clear relationship between the displacement of the reference centroid and initial motion phase was observed from the analysis. However, the following were key findings: 1) total vector centroid displacements as large as 11 mm, typically in the longitudinal (S-I) direction, were possible for the fast scan techniques, 2) centroid misplacement for the slow-scan technique was greater in the transaxial (AP and L-R) directions with misplacement magnitudes as much as 11 mm, and 3) centroid misrepresentation was greater for the smaller target.

**Table 5:** Range of misrepresentation of the centroid of the 10- and 31.5-mm diameter targets.

Target Diameter (mm)	S-I Motion Amplitude ± (mm)	Centroid mis-placement (mm), single-slice fast scan CT					
		
		S-I		A-P		R-L	
		
		Mean	95% CI	Mean	95% CI	Mean	95% CI
10	5	-0.8	6.2	0.2	1.0	0.6	1.2
	10	-1.1	5.2	0.3	2.2	0.7	1.8
	15	-0.7	7.0	0.2	1.4	1.6	3.0

31.5	5	-2.2	5.2	-0.2	0.2	-0.1	0.6
	10	-2.7	4.2	-0.3	1.4	0.4	1.6
	15	-2.5	8.8	-0.5	1.0	0.1	1.2

Target Diameter (mm)	S-I Motion Amplitude ± (mm)	Centroid mis-placement (mm), multi-slice fast scan CT					
		
		S-I		A-P		R-L	
		
		Mean	95% CI	Mean	95% CI	Mean	95% CI

10	5	1.1	4.6	0.0	1.4	0.5	0.6
	10	0.4	3.0	0.0	1.4	0.4	1.2
	15	0.4	5.4	0.1	1.6	0.2	1.0

31.5	5	0.5	3.4	0.3	0.8	0.7	0.8
	10	1.2	5.4	0.1	1.0	1.1	1.2
	15	2.0	5.4	-0.4	0.8	0.8	1.2

Target Diameter (mm)	S-I Motion Amplitude ± (mm)	Centroid mis-placement (mm), single-slice slow scan CT					
		
		S-I		A-P		R-L	
		
		Mean	95% CI	Mean	95% CI	Mean	95% CI

10	5	-0.2	1.8	1.2	2.4	2.4	2.8
	10	-0.9	2.6	3.3	1.4	4.2	1.8
	15	-1.3	1.4	5.9	3.6	7.1	3.6

31.5	5	-2.2	3.4	0.0	1.0	1.3	3.6
	10	-1.8	2.6	0.0	2.4	0.8	2.2
	15	-2.8	2.4	0.9	3.4	1.6	2.4

From top to bottom, the tables were generated from single-slice fast (or 1-sec), multi-slice fast (or 1-sec), and single-slice slow (or 4-sec) scan acquisition techniques, respectively. Ranges were computed for three known motion amplitudes.

## Discussion

Virtual radiation therapy simulation for lung and abdominal targets typically relies on intermediate-rotational-speed, helical (or spiral) CT for target volume localization. Most helical CT simulator units, including the ones used for data acquisition in the present study, are capable of acquiring images at rotational speeds between 1 and 4 seconds. Slow image acquisition rotation speeds are not necessarily available for all dedicated devices. The benefit of increased volume coverage with helical CT comes with the price tag, in the presence of physiologic motion, of increased data inconsistency.

### Helical CT

During helical CT data acquisition, there exists simultaneous gantry (x-ray tube and detector system) rotation with continuous table feed. Furthermore, CT projection data are a measure of the integral absorption along fan beam lines for all views during (full) gantry rotation. Similar to axial CT, every subsequent view is acquired at a different angle. However, in helical CT, the longitudinal position of a view with respect to the imaged object changes constantly, depending on the preset scan pitch. Under these circumstances, projections are not collected on a slice-by-slice basis. Projections for each corresponding slice are reconstructed by suitable interpolation between adjacent projections.

### 3D target motion

Image reconstruction in helical CT is optimized with the premise that imaged objects are stationary. However, tumors are not always stationary, especially those located in the thorax or abdomen, which typically exhibit periodic 3D motion. In such instances, the targets' cross section and position in the imaging plane varies continuously as it moves into or out of, as well as within, the imaging plane. In this study, a spherical target geometry was used. The diameter registered by each subsequent view increases or decreases, depending on the target motion phase. Thus, as the plane of reconstruction changes, views from different longitudinal positions in the target are used for interpolation, hence, influencing the orientation of the geometry of the reconstructed target.

### Motion-induced artifacts

Unlike planar x-ray imaging, where target motion leads to blurring, or averaging, based on the extent and type of motion, motion-induced artifacts in CT imaging arise from the fact that moving objects are at different locations at different projection angles. During the helical acquisition methodology, the motion induced artifacts are also influenced by the slice acquisition time, the temporal relationship between data acquisition and target motion cycle, and the initial angle of the x-ray source. There are numerous publications in the literature describing techniques to eliminate or, at least, minimize motion-induced artifacts, but these very interesting works are beyond the scope of this work. In the present study, true three-dimensional target motion resembling more closely a clinically observed target motion pattern, albeit an idealized or a simplified model, was investigated. Despite differences in study design, the results of the present study can be partially compared with findings in the literature [2,3,6].

### Fast (1-s)-scan helical CT

**Figure 6:**
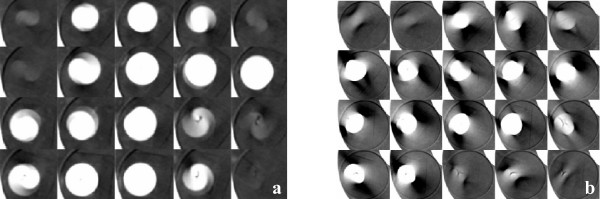
Sequential axial slices for the 31.5-mm diameter target. Images were reconstructed from a fast-scan (a) and slow-scan (b) of the target to illustrate the effect of motion on data projection.

During the fast-scan CT technique for a target moving in/out and within the imaging plane, a finite but small number of different phases of target motion are partially projected within the image plane resulting in misrepresentation of target cross section as is shown in Fig. 6a. It should be noted that the target cross section is not a disc with a uniform CT number, as might be expected. Furthermore, the reconstructed intensities from projections from the S-I poles of the targets are underweighted, whereas those in the middle are over-weighted. Motion-induced artifacts occur in small and large targets alike; however, smaller targets are more susceptible to geometric misses. When motion amplitude is larger than target diameter, the probability of a target moving completely out of the imaging plane, and hence, being "not seen' by a view, is greater [9]. While there may appear to be a pattern of some sort in Fig 3, this would not imply that *a priori *knowledge of target geometry and of motion and CT parameters will lead to dGTV prediction.

Recently, a carefully designed experiment- and simulation-based review on motion-induced artifacts as a function of fast-scan CT acquisition techniques (i.e., short slice acquisition times relative to motion cycle periods), was reported by Chen and colleagues [2] from Massachusetts General Hospital (MGH). The authors concluded that distortions along the axis of motion could result in either a lengthening or shortening of the target. In addition to shape distortion, the center of the imaged target can be displaced by as much as the amplitude of the motion, similar to findings in the present study (Fig 5). However, there were some notable differences in their findings and findings in the current study. While the MGH group reported both overestimations and underestimations of moving target volumes, as did Caldwell and colleagues [3] and Kini and colleagues [7], only in one instance was a slight amount of underestimation observed in the present study. This was an interesting variation in findings, which may be attributable to several subtle, but important, technical differences in the methods utilized in these related studies. Studies by both Caldwell and Kini characterized the ratio of dynamically-imaged-target-volume, referred to as dGTV in this study, over the target volume derived from a static image (here referred to as sGTV). This differs from the ratio reported in our study, namely dGTV/tTV (or true Target Volume as measured directly from the object). Given that Weiner-Muram and colleagues [1] have shown that CT volume averaging effects of imaging static 10- and 31.5-mm diameter objects with a 3 mm CT slice thickness can result in over-estimation of the true target volumes by as much as 40% and 12%, respectively, it is not difficult to understand that the ratio reported by Caldwell and Kini, with a larger sGTV representation of the true target volume in the denominator, might be smaller than that observed in the present study where the *true *measured target volume was used in the denominator. Regarding the MGH group findings, it is important to understand that their work represented a computer simulation study which characterized the time-varying geometric intersection of a CT slice dimension with a moving object and, as such, did not seek to attain Hounsfield number representations of the resulting image. While valuable in helping to characterize the geometric misrepresentations of shape and position which can result from CT imaging of moving objects, this study did not attempt to quantify the variation in dGTV in the same way that this term is defined here.

An additional contributing factor to the lower percentage of volume under-estimation observed in this study, relative to the Caldwell and colleagues study, is that the Caldwell group stated that the Hounsfield unit threshold used to define dGTV borders was determined by systematically matching the geometry of the sGTV with its physical values while at the same time excluding in-air CT image artifacts. This would likely have required an increasing of the window level settings, which would have subsequently reduced the volume of visible dGTV, relative to our method, which did not force such agreement between the sGTV and tTV. Such an approach would have further contributed to the noted differences between this study and the Caldwell and colleagues study.

A final, and likely, contributing factor to the differences in volume underestimation observed by our study, relative to the previously mentioned studies, would be the presence in our study of 3D target motion. The addition of volume aliasing effects in the axial plane, second to target motion in this plane, would certainly contribute to a growth in the dGTV volumes that we measured. In light of the fact that the previously mentioned studies utilized linear motion, absent of axial-plane translations, it is understandable that this increase in value of the dGTV (numerator) value of the reported ratio would lead to a further reduction of volume underestimation observed by our study.

While the 3D sinusoidal model used here to approximate a complex human respiratory cycle is clearly a simplification, it is still (arguably) a reasonable experimental compromise in the representation of the magnitude of tumor aliasing errors introduced when imaging moving targets. The failure of the phantom to accurately model the natural and characteristic pause at the end of the normal human exhale cycle may well result in somewhat of an *overestimation *of the target *volume *aliasing, relative to a true human breathing cycle, since the phantom target will not pause and, thus, not afford the scanner the opportunity to capture at least part of its image in a relatively motionless state. In contrast however, the simplified sinusoidal model will likely *underestimate *the potential to misrepresent the *centroid *location of the moving target, relative to a true human breathing cycle, due to its failure to model the very same pause at the end of the exhale cycle, which subsequently affords the scanner the increased probability of capturing an image of a real, human tumor at a point located at its maximum distance from the central position.

### Slow (4-s)-scan helical CT

**Figure 7:**
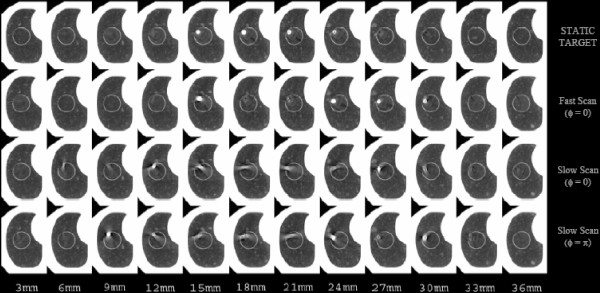
Four CTs of the phantom with the embedded 10 mm diameter spherical target. Each image in the series represents a 3-mm transaxial reconstruction of helically acquired CT data. The first series (STATIC TARGET) depicts image acquisition with the target stationary, and serves as a reference and a surrogate of the true axial geometry of the imaged target. The second series depicts the same target scanned with a slice acquisition time of 1 s and moving in 3D. The third and fourth series illustrate the effect of changing slice acquisition time from 1 s to 4 seconds (acquisitions in series 3) and also changing the initial motion-to-scan phase relationships from 0 to π(acquisitions in series 4).

**Figure 8:**
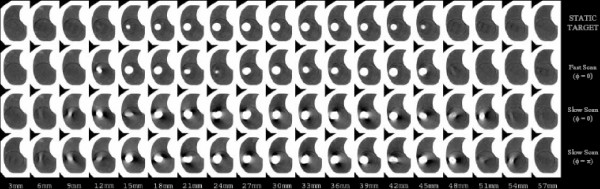
Four CT studies of the phantom with the embedded 3.15 cm diameter spherical target. Each image in the series represents a 3-mm transaxial reconstruction of helically acquired CT data. The first series (STATIC TARGET) depicts image acquisition with the target stationary, and serves as a reference and a surrogate of the true axial geometry of the imaged target. The second series depicts the same target scanned with a 1-s slice acquisition time and moving in 3D. The third and fourth series illustrate the effect of changing slice acquisition time from 1 s to 4 seconds (acquisitions in series 3) and also changing the initial motion-to-scan phase relationships from 0 to π(acquisitions in series 4).

Wurstbauer and colleagues [6] recently showed that slow-scan acquisition CTs result in larger, but highly constant depictions of lung tumors in comparison to fast-scan techniques, yielding an integral delineation of almost all positions of the moving tumors. The authors concluded that the use of slow planning CTs enables the drawing of tighter margins in external beam treatment planning of lung cancer. Theoretically, slow-scan techniques with slice acquisition times equal to or greater than the period of target motion should detect the range of tumor motion and shape throughout the normal motion cycle. However, as shown in Fig 4, aliasing errors still exist in the reconstructed projection data. While slow-scan techniques generate target volumes larger than fast-scan target volumes, and while slow-scan generated images appear to be more reproducible and seem to approximate the time-average motion profile [10,11], this was shown true only from analytical/simulation studies. Findings in this study showed a perceptible dependence of reconstructed volume on the temporal relationship between initial target motion-phase and initial angle of x-ray source, as illustrated in as well as Fig 7 and 8 (acquisitions in series 3 and 4) for two different initial motion phases. Once again, as the plane of reconstruction changes, different views are used for helical interpolation. The direction of these views thus determines the orientation of the reconstructed target geometry.

Unlike the fast-scan reconstructed images where target-to-normal-tissue interfaces may be more discernible, images from the slow-scan technique demonstrate shading artifacts (Fig 6b and 7). While a finite, but small, number of different phases of target motion are partially projected within the image plane during the fast scan technique, a finite, but large, number are projected during the slow scan technique. Thus, many more completely different longitudinal positions of the moving target are used for projection reconstruction during slow scanning; hence, inconsistencies in the views result in significant shading artifacts.

Finally, while significant deviations were observed in S-I centroids of dGTVs of the fast-scan acquisitions, such deviation were observed in the *transaxial *(A-P and R-L) directions for the slow-scan technique (Fig 5). This is due, in part, to the significant influence of longitudinal motion on fast-scan acquisitions, and both longitudinal and transversal motion on slow-scan acquisitions, when the target is being frozen in time. In theory, it is more likely for the centroid of a slow-scan dGTV to coincide with its corresponding reference centroid. However, this is rarely the case in reality due to the complexity of the data acquisition process.

### A note on slice thickness

**Figure 9:**
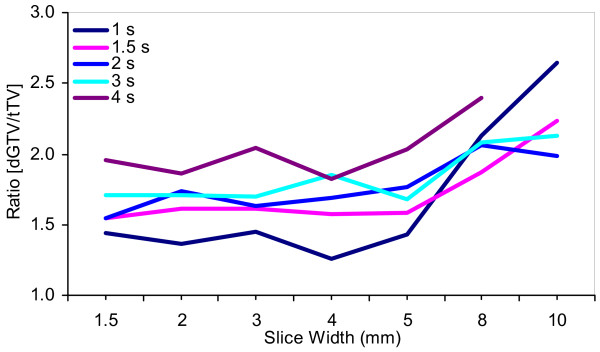
Magnitude of mis-estimation of the true physical volumes (tTV) of the 31.5-mm diameter target as a function of slice width for five (5) different CT slice acquisition times. The mis-estimation magnitude is computed as a ratio of each CT reconstructed dGTV and its corresponding tTV. Geometric variation is illustrated for the reference initial motion-to-scan phase relationship only.

Despite the fact that the slice widths used in the present study (2.5 and 3 mm for the single-slice and multi-slice techniques, respectively) are not fully-encompassing of existing clinical practices, it is worth noting that increasing slice thickness not only changes the reconstructed dGTV, but can potentially increase it (Fig 9). This is attributable, in part, to increasing partial volume averaging in the longitudinal direction, similar to findings by Winer-Muram and colleagues [1] on static targets.

## Conclusions and clinical implications

Accurate appreciation and delineation of target volume in radiation oncology is not only crucial for designing an appropriate and clinically effective treatment plan, but also necessary for accurate dose calculation. Understanding and fully characterizing potential errors caused by target motion is a complex subject that will require future characterization. Phantom studies such as the present study using a dynamic anthropomorphic thorax phantom provide an approximation of the impact of 3D target motion as a function of specific scan parameters chosen for pre-treatment planning CT data acquisition. The present data support one key conclusion during non-gated CT acquisitions: when using a single-slice spiral CT, slow scanning image acquisition appears to be the most practical method of acquiring data that may (more) reliably characterize the time-average position and shape of a moving target. Fast scanning on such scanners, as well as on a 4-slice multi-slice scanner, on the other hand, provides for more reliably definition of the boundary between target and normal tissue. Or, stated conversely, non-gated spiral CT imaging of dynamic targets, can lead not only to distortions of target shape and misplacement of target centroid, but also to complete loss of volumetric information, as well as information about the motion of the target. The impact of image misrepresentation and loss of viable target information introduces a systematic error in designed treatment plan, and hence, a potential source of dose misrepresentation. While the present study did not take into account target deformation, which would have further complicated interpretation of result in this study, it is clear that utilization of population-based planning target volumes might not serve all patients well in that, even for patients with identical breathing patterns, there are still significant individual variations in the extent of deformation of dGTV as a result of variable motion phase. In any case, a failure in accurate localization and characterization of target geometry during free breathing, non-gated CT imaging imposes a limitation on the therapeutic gains of conformally implemented RT techniques.

## Competing interests

V. Varchena is an employee of CIRS Inc., which has commercialized the dynamic anthropomorphic thorax phantom.

## Authors' contributions

JAT participated in the conception and design of the study, carried out the data acquisition, performed data analysis, evaluated the results and drafted the manuscript. MF participated in the design of the study and revised the manuscript. VV participated in the design of the study, performed modifications of the phantom relevant to the conduct of the study, and revised the manuscript. JLL participated in the design of the study and revised the manuscript. BJS participated in the conception and design of the study, interpretation of data and drafting of the manuscript. All authors read and approved the final manuscript.

## Appendix

The path described by the centroid of the equatorial slice of the target about the central axis of the target-adapted rod can be modeled by the parametric equation (with rectangular coordinates) s [x(t), y(t), z(t)] where s(t)=[hcos⁡(at),hsin⁡(at),b2t]
 MathType@MTEF@5@5@+=feaafiart1ev1aaatCvAUfKttLearuWrP9MDH5MBPbIqV92AaeXatLxBI9gBaebbnrfifHhDYfgasaacH8akY=wiFfYdH8Gipec8Eeeu0xXdbba9frFj0=OqFfea0dXdd9vqai=hGuQ8kuc9pgc9s8qqaq=dirpe0xb9q8qiLsFr0=vr0=vr0dc8meaabaqaciaacaGaaeqabaqabeGadaaakeaacqWGZbWCcqGGOaakcqWG0baDcqGGPaqkcqGH9aqpdaWadaqaaiabdIgaOjGbcogaJjabc+gaVjabcohaZjabcIcaOiabdggaHjabdsha0jabcMcaPiabcYcaSiabdIgaOjGbcohaZjabcMgaPjabc6gaUjabcIcaOiabdggaHjabdsha0jabcMcaPiabcYcaSmaalaaabaGaemOyaigabaGaeGOmaidaaiabdsha0bGaay5waiaaw2faaaaa@4D96@

where *h *is the distance from the centroid of the target to the axis of rotation of the target adapted rod, *a *is the angular rotation speed (radians/sec) of the target centroid around the central axis of the target adapted rod, *b *is the S-I amplitude, and (*b/2*) defines the speed of target motion, and *t *is a time function. Let L be the arc length of the path described by the centroid of the target. Relevant parameters used to determine benchmark volumes are summarized in Table 6.

**Table 6:** Relevant parameters used to determine benchmark volumes tTV and tGTV.

Target Diameter (mm)	*a* (radians/sec)	*b* (mm)	*t* (s)	*h* (mm)
10	π/4	10, 20, 30	0 to 2	14.25
31.5	39.4π/180	10, 20, 30	0 to 2	15.75

L=∫0t(dxdt)2+(dydt)2+(dzdt)2dt'
MathType@MTEF@5@5@+=feaafiart1ev1aaatCvAUfKttLearuWrP9MDH5MBPbIqV92AaeXatLxBI9gBaebbnrfifHhDYfgasaacH8akY=wiFfYdH8Gipec8Eeeu0xXdbba9frFj0=OqFfea0dXdd9vqai=hGuQ8kuc9pgc9s8qqaq=dirpe0xb9q8qiLsFr0=vr0=vr0dc8meaabaqaciaacaGaaeqabaqabeGadaaakeaacqWGmbatcqGH9aqpdaWdXbqaamaakaaabaWaaeWaaeaadaWcaaqaaiabdsgaKjabdIha4bqaaiabdsgaKjabdsha0baaaiaawIcacaGLPaaadaahaaWcbeqaaiabikdaYaaakiabgUcaRmaabmaabaWaaSaaaeaacqWGKbazcqWG5bqEaeaacqWGKbazcqWG0baDaaaacaGLOaGaayzkaaWaaWbaaSqabeaacqaIYaGmaaGccqGHRaWkdaqadaqaamaalaaabaGaemizaqMaemOEaOhabaGaemizaqMaemiDaqhaaaGaayjkaiaawMcaamaaCaaaleqabaGaeGOmaidaaaqabaGccqWGKbazcqWG0baDcqGGNaWjaSqaaiabicdaWaqaaiabdsha0bqdcqGHRiI8aaaa@51F7@

d(s(t))dt=[−ahsin⁡(at),ahcos⁡(at),b2]
 MathType@MTEF@5@5@+=feaafiart1ev1aaatCvAUfKttLearuWrP9MDH5MBPbIqV92AaeXatLxBI9gBaebbnrfifHhDYfgasaacH8akY=wiFfYdH8Gipec8Eeeu0xXdbba9frFj0=OqFfea0dXdd9vqai=hGuQ8kuc9pgc9s8qqaq=dirpe0xb9q8qiLsFr0=vr0=vr0dc8meaabaqaciaacaGaaeqabaqabeGadaaakeaadaWcaaqaaiabdsgaKnaabmaabaGaem4CamNaeiikaGIaemiDaqNaeiykaKcacaGLOaGaayzkaaaabaGaemizaqMaemiDaqhaaiabg2da9maadmaabaGaeyOeI0IaemyyaeMaemiAaGMagi4CamNaeiyAaKMaeiOBa4MaeiikaGIaemyyaeMaemiDaqNaeiykaKIaeiilaWIaemyyaeMaeeiAaGMagi4yamMaei4Ba8Maei4CamNaeiikaGIaemyyaeMaemiDaqNaeiykaKIaeiilaWYaaSaaaeaacqWGIbGyaeaacqaIYaGmaaaacaGLBbGaayzxaaaaaa@5552@

L=∫02(−ahsin⁡(at))2+(ahcos⁡(at))2+(b2)2dt'
MathType@MTEF@5@5@+=feaafiart1ev1aaatCvAUfKttLearuWrP9MDH5MBPbIqV92AaeXatLxBI9gBaebbnrfifHhDYfgasaacH8akY=wiFfYdH8Gipec8Eeeu0xXdbba9frFj0=OqFfea0dXdd9vqai=hGuQ8kuc9pgc9s8qqaq=dirpe0xb9q8qiLsFr0=vr0=vr0dc8meaabaqaciaacaGaaeqabaqabeGadaaakeaacqWGmbatcqGH9aqpdaWdXbqaamaakaaabaGaeiikaGIaeyOeI0IaemyyaeMaemiAaGMagi4CamNaeiyAaKMaeiOBa4MaeiikaGIaemyyaeMaemiDaqNaeiykaKIaeiykaKYaaWbaaSqabeaacqaIYaGmaaGccqGHRaWkcqGGOaakcqWGHbqycqWGObaAcyGGJbWycqGGVbWBcqGGZbWCcqGGOaakcqWGHbqycqWG0baDcqGGPaqkcqGGPaqkdaahaaWcbeqaaiabikdaYaaakiabgUcaRmaabmaabaWaaSaaaeaacqWGIbGyaeaacqaIYaGmaaaacaGLOaGaayzkaaWaaWbaaSqabeaacqaIYaGmaaaabeaakiabdsgaKjabdsha0jabcEcaNaWcbaGaeGimaadabaGaeGOmaidaniabgUIiYdaaaa@5AA2@

L=∫02(ah)2(sin⁡2(at)+cos⁡2(at))+(b2)2dt'
MathType@MTEF@5@5@+=feaafiart1ev1aaatCvAUfKttLearuWrP9MDH5MBPbIqV92AaeXatLxBI9gBaebbnrfifHhDYfgasaacH8akY=wiFfYdH8Gipec8Eeeu0xXdbba9frFj0=OqFfea0dXdd9vqai=hGuQ8kuc9pgc9s8qqaq=dirpe0xb9q8qiLsFr0=vr0=vr0dc8meaabaqaciaacaGaaeqabaqabeGadaaakeaacqWGmbatcqGH9aqpdaWdXbqaamaakaaabaGaeiikaGIaemyyaeMaemiAaGMaeiykaKYaaWbaaSqabeaacqaIYaGmaaGcdaqadaqaaiGbcohaZjabcMgaPjabc6gaUnaaCaaaleqabaGaeGOmaidaaOGaeiikaGIaemyyaeMaemiDaqNaeiykaKIaey4kaSIagi4yamMaei4Ba8Maei4Cam3aaWbaaSqabeaacqaIYaGmaaGccqGGOaakcqWGHbqycqWG0baDcqGGPaqkaiaawIcacaGLPaaacqGHRaWkdaqadaqaamaalaaabaGaemOyaigabaGaeGOmaidaaaGaayjkaiaawMcaamaaCaaaleqabaGaeGOmaidaaaqabaGccqWGKbazcqWG0baDcqGGNaWjaSqaaiabicdaWaqaaiabikdaYaqdcqGHRiI8aaaa@5811@

sin^2^(*at*)+cos^2^(*at*) = 1

L=∫02(ah)2+(b2)2dt'
MathType@MTEF@5@5@+=feaafiart1ev1aaatCvAUfKttLearuWrP9MDH5MBPbIqV92AaeXatLxBI9gBaebbnrfifHhDYfgasaacH8akY=wiFfYdH8Gipec8Eeeu0xXdbba9frFj0=OqFfea0dXdd9vqai=hGuQ8kuc9pgc9s8qqaq=dirpe0xb9q8qiLsFr0=vr0=vr0dc8meaabaqaciaacaGaaeqabaqabeGadaaakeaacqWGmbatcqGH9aqpdaWdXbqaamaakaaabaGaeiikaGIaemyyaeMaemiAaGMaeiykaKYaaWbaaSqabeaacqaIYaGmaaGccqGHRaWkdaqadaqaamaalaaabaGaemOyaigabaGaeGOmaidaaaGaayjkaiaawMcaamaaCaaaleqabaGaeGOmaidaaaqabaGccqWGKbazcqWG0baDcqGGNaWjaSqaaiabicdaWaqaaiabikdaYaqdcqGHRiI8aaaa@4226@

L=2(ah)2+(b2)2
 MathType@MTEF@5@5@+=feaafiart1ev1aaatCvAUfKttLearuWrP9MDH5MBPbIqV92AaeXatLxBI9gBaebbnrfifHhDYfgasaacH8akY=wiFfYdH8Gipec8Eeeu0xXdbba9frFj0=OqFfea0dXdd9vqai=hGuQ8kuc9pgc9s8qqaq=dirpe0xb9q8qiLsFr0=vr0=vr0dc8meaabaqaciaacaGaaeqabaqabeGadaaakeaacqWGmbatcqGH9aqpcqaIYaGmdaGcaaqaaiabcIcaOiabdggaHjabdIgaOjabcMcaPmaaCaaaleqabaGaeGOmaidaaOGaey4kaSYaaeWaaeaadaWcaaqaaiabdkgaIbqaaiabikdaYaaaaiaawIcacaGLPaaadaahaaWcbeqaaiabikdaYaaaaeqaaaaa@3B2D@

The time-averaged volume traced by the target can be computed using

V=π(r‖N‖)2L+43π r3
 MathType@MTEF@5@5@+=feaafiart1ev1aaatCvAUfKttLearuWrP9MDH5MBPbIqV92AaeXatLxBI9gBaebbnrfifHhDYfgasaacH8akY=wiFfYdH8Gipec8Eeeu0xXdbba9frFj0=OqFfea0dXdd9vqai=hGuQ8kuc9pgc9s8qqaq=dirpe0xb9q8qiLsFr0=vr0=vr0dc8meaabaqaciaacaGaaeqabaqabeGadaaakeaacqWGwbGvcqGH9aqpiiGacqWFapaCdaqadaqaaiabdkhaYnaafmaabaacbaGae4Nta4eacaGLjWUaayPcSdaacaGLOaGaayzkaaWaaWbaaSqabeaacqaIYaGmaaGccqWGmbatcqGHRaWkdaWcaaqaaiabisda0aqaaiabiodaZaaacqWFapaCcqqGGaaicqWGYbGCdaahaaWcbeqaaiabiodaZaaaaaa@4224@

where *r *is the radius of the target, ||N|| is a unit normal vector defining the perpendicularity of the radius of the equatorial slice to its path.

V=2π r2(ah)2+(b2)2+43π r3
 MathType@MTEF@5@5@+=feaafiart1ev1aaatCvAUfKttLearuWrP9MDH5MBPbIqV92AaeXatLxBI9gBaebbnrfifHhDYfgasaacH8akY=wiFfYdH8Gipec8Eeeu0xXdbba9frFj0=OqFfea0dXdd9vqai=hGuQ8kuc9pgc9s8qqaq=dirpe0xb9q8qiLsFr0=vr0=vr0dc8meaabaqaciaacaGaaeqabaqabeGadaaakeaacqWGwbGvcqGH9aqpcqaIYaGmiiGacqWFapaCcqqGGaaiieGacqGFYbGCdaahaaWcbeqaaiabbkdaYaaakmaakaaabaGaeiikaGIaemyyaeMaemiAaGMaeiykaKYaaWbaaSqabeaacqaIYaGmaaGccqGHRaWkdaqadaqaamaalaaabaGaemOyaigabaGaeGOmaidaaaGaayjkaiaawMcaamaaCaaaleqabaGaeGOmaidaaaqabaGccqGHRaWkdaWcaaqaaiabisda0aqaaiabiodaZaaacqWFapaCcqqGGaaicqGFYbGCdaahaaWcbeqaaiabbodaZaaaaaa@4848@

## References

[B1] Winer-Muram HT, Jennings SG, Meyer CA, Liang Y, Aisen A, Tarver RD, McGarry RC (2003). Effect of varying CT section with on volumetric measurement of lung tumors and application of compensatory equations. Radiology.

[B2] Chen GTY, Kung JH, Beaudette KP (2004). Artifacts in computed tomography scanning of moving objects. Semin Radiat Oncol.

[B3] Caldwell CB, Mah K, Skinner M, Danjoux CE (2003). Can PET provide the 3D extent of tumor motion for individualized internal target volumes? A phantom study of the limitations of CT and the promises of PET. Int J Radiat Oncol Biol Phys.

[B4] Ozhasoglu C, Murphy MJ (2002). Issues in respiratory motion compensation during external-beam radiotherapy. Int J Radiat Oncol Biol Phys.

[B5] Langen KM, Jones DJ (2001). Organ motion and its management. Int J Radiat Oncol Biol Phys.

[B6] Wurstbauer K, Deutschmann H, Kopp P, Sedlmayer F (2005). Radiotherapy planning for lung cancer: slow CTs allow the drawing of tighter margins. Radiother Oncol.

[B7] Kini VR, Keall PJ, Vedam SS, Arthur DW, Kavanagh BD, Cardinale RM, Mohan R (2000). Preliminary results from a study of a respiratory motion tracking system: Underestimation of target volume with conventional CT simulation. Int J Radiat Oncol Biol Phys.

[B8] Van Hoe L, Haven F, Bellon E, Baert AL, Bosmans H, Feron M, Suetens P, Marchal G (1997). Factors Influencing the Accuracy of Volume Measurements in Spiral CT: A Phantom Study. J Comput Assist Tomogr.

[B9] Wilting JE, Timmer J (1999). Artefacts in spiral-CT images and their relation to pitch and subject morphology. Eur Radiology.

[B10] Lagerwaard FJ, Van Sörnsen de Koste JR, Nijssen-Visser MRJ, Schuchhard-Schipper RH, Oei SS, Munne A, Senan S (2001). Multiple "slow" CT scans for incorporating lung tumor mobility in radiotherapy planning. Int J Radiat Oncol Biol Phys.

[B11] van Sörnsen de Koste JR, Lagerwaard FJ, de Boer HCJ, Nijssen-Visser MR, Senan S (2003). Are multiple CT scans required for planning curative radiotherapy in lung tumors of the lower lobe?. Int J Radiat Oncol Biol Phys.

